# Evolutionary trends in antifungal resistance: a meta-analysis

**DOI:** 10.1128/spectrum.02127-23

**Published:** 2024-03-06

**Authors:** Xueke Niu, Abdullah M. S. Al-Hatmi, Roxana G. Vitale, Michaela Lackner, Sarah A. Ahmed, Paul E. Verweij, Yingqian Kang, Sybren de Hoog

**Affiliations:** 1 Key Laboratory of Environmental Pollution Monitoring and Disease Control, Ministry of Education of Guizhou & Key Laboratory of Microbiology and Parasitology of Education Department of Guizhou, School of Basic Medical Sciences, Guizhou Medical University, Guiyang, China; 2 Center of Expertise in Mycology of Radboud University Medical Center/Canisius Wilhelmina Hospital, Nijmegen, The Netherlands; 3 Natural & Medical Science Research Center, University of Nizwa, Nizwa, Oman; 4 Consejo Nacional de Investigaciones Científicas y Tecnológicas (CONICET), Buenos Aires, Argentina; 5 Unidad de Parasitología, Sector Micología, Hospital J.M. Ramos Mejía, Buenos Aires, Argentina; 6 Institute of Hygiene and Medical Microbiology, Medical University of Innsbruck, Innsbruck, Austria; University of Guelph, Guelph, Ontario, Canada

**Keywords:** antifungals, resistance, breakpoint, phylogeny

## Abstract

The present paper includes a meta-analysis of literature data on 318 species of fungi belonging to 34 orders in their response to 8 antifungal agents (amphotericin B, caspofungin, fluconazole, itraconazole, ketoconazole, posaconazole, terbinafine, and voriconazole). Main trends of MIC results at the ordinal level were visualized. European Committee on Antimicrobial Susceptibility Testing and Clinical & Laboratory Standards Institute (CLSI) clinical breakpoints were used as the staff gauge to evaluate MIC values ranging from resistance to susceptibility, which were subsequently compared with a phylogenetic tree of the fungal kingdom. Several orders (*Hypocreales, Microascales*, and *Mucorales*) invariably showed resistance. Also the basidiomycetous orders *Agaricales, Polyporales, Sporidiales, Tremellales*, and *Trichosporonales* showed relatively high degrees of azole multi-resistance, while elsewhere in the fungal kingdom, including orders with numerous pathogenic and opportunistic species, that is, *Onygenales, Chaetothyiales, Sordariales*, and *Malasseziales*, in general were susceptible to azoles. In most cases, resistance vs susceptibility was consistently associated with phylogenetic distance, members of the same order showing similar behavior.

**IMPORTANCE:**

A kingdom-wide the largest set of published wild-type antifungal data comparison were analyzed. Trends in resistance in taxonomic groups (monophyletic clades) can be compared with the phylogeny of the fungal kingdom, eventual relationships between fungus–drug interaction and evolution can be described.

## INTRODUCTION

Infections by fungi tend to be chronic and recalcitrant with frequent relapse, and hence management can be problematic in compromised patients and sometimes even in otherwise healthy individuals. Susceptibility of human cohorts is increasing already for decades due to widespread constitutional factors such as diabetes, transplant, and leukemia (1–5). The Leading International Fungal Education website estimates that >80% of the world's population suffer from any kind of fungal infection (6). The species spectrum of proven infections today, including pathogens and opportunists, comprises over 720 agents (7). Although the majority of infections is caused by a limited number of human-associated pathogens such as *Candida*, dermatophytes, and relatives of *Histoplasma*, a significant share is due to a gamut of environmental fungi (8–10). The most frequent opportunist by far is *Aspergillus fumigatus*, a saprobe on decomposing plant material (11). Recent reports estimate the occurrence of around 3 million cases of chronic pulmonary aspergillosis today (12). Some fungal groups, particularly in Mucorales, are able to cause rapidly progressive, acute invasive infections in susceptible patients, such as rhinocerebral mucormycosis, a life-threatening condition that requires immediate medical attention, the disease becoming rapidly fatal in 50%–80% of patients if left untreated (13–15). Other potentially fatal conditions include *Cryptococcus neoformans* in individuals with impaired acquired immunity, especially in AIDS, with an estimation of over a million cases per year and with 60% of these patients dying within 3  months after diagnosis (16).

Antifungal drug resistance (17–19) in fungal opportunists is an increasing problem in modern medicine (20). Especially the rapid emergence of multi-drug resistant (MDR) species such as *Candida auris* (21) and Covid-19-associated mucormycosis have challenged hospital hygiene measures and patient management. The chronic nature of fungal infections poses a severe management problem with immunocompromised patients, and sometimes even in otherwise healthy individuals. The emergence of drug- and MDR species has been linked to environmental exposure of saprobic fungi to short-tailed azole fungicides (22–24). Such drugs, developed for the protection of agricultural crops, are used in the dimension of gigatons in monocultures, factory farming, aquaculture, mass-agriculture, and in shipping industries (25, 26). Subsequent to wide-spread application, azoles accumulate in soils and water bodies (24). Short-tailed agricultural azoles such as benomyl, carbendazim, flubendazole, imazalil, oxpoconazole, triflumizole, diniconazole, epoxiconazole, and flutriafol are similar in chemical structure to compounds used in human medicine (23, 27, 28). Environmental azole contamination leads to a selective pressure toward azole-resistance in saprobic and opportunistic fungi which may reappear as etiologic agents of disease in human medicine (23, 29, 30). Looking at this problem from a one-health perspective, separation of compound classes used in human medicine vs agriculture is overdue.

Antifungal resistance, as defined in the guidelines of the European Committee on Antimicrobial Susceptibility Testing (EUCAST), can be classified as microbiological vs clinical (31, 32), referring to intrinsic abilities of the fungus vs recalcitrance of the infection. Resistance may involve a single compound, but more often multi-resistance is observed against different antifungal classes. Resistance is mostly described for particular species, such as *C. krusei*, *C. auris*, or *Naganishia albida,* even though several entire genera or families may show multi-resistance. Consequently, multi-resistant genera are often phylogenetically related, as members of a single order. Examples are *Acremonium–Fusarium–Trichoderma* belonging to the *Hypocreales*, and *Scedosporium–Lomentospora–Microascus–Scopulariopsis* belonging to the *Microascales*. Consequently, the phylogenetic position of a fungus predicts its antifungal resistance. This demonstrates a certain relationship between phylogeny and resistance, which we aim to explore in the present manuscript.

The commonly systemically applied antifungal agents belong to four classes: azoles, polyenes, allylamines, and echinocandins. With systemic and disseminated infections, susceptibility testing is done in clinical settings on patient isolates to provide approximate guidance for management (7). For some groups, such as *Aspergillus* and *Candida,* results can be validated with clinical breakpoints (CBPs) (33), which guide to a reliable estimate of the therapeutic sensitivity of the fungus. However, for the great majority of fungal opportunists, no CBPs have been determined, and approximate evaluation has to be done by analogy with phylogenetically remote species (21, 34).

A kingdom-wide comparison of antifungal susceptibility has been enabled by the new *Atlas of Clinical Fungi* (7), where the largest set of published wild-type antifungal data has been brought together. Trends in resistance in taxonomic groups (monophyletic clades) can be compared with the phylogeny of the fungal kingdom, which is the aim of the present paper. Through a comparative analysis of multiple groups and across groups, consistent behavior, and changeability in resistance can be detected and quantified, which provides a certain degree of therapeutic predictivity for the particular etiologic agent. Eventual relationships between fungus–drug interaction and evolution can be described. Main functional roles in drug resistance mechanisms and fungal evolution may be revealed, as a cornerstone for future research on the significance of antifungal resistance in nature vs the clinic.

## MATERIALS AND METHODS

### Data collection

**Fig 1:**
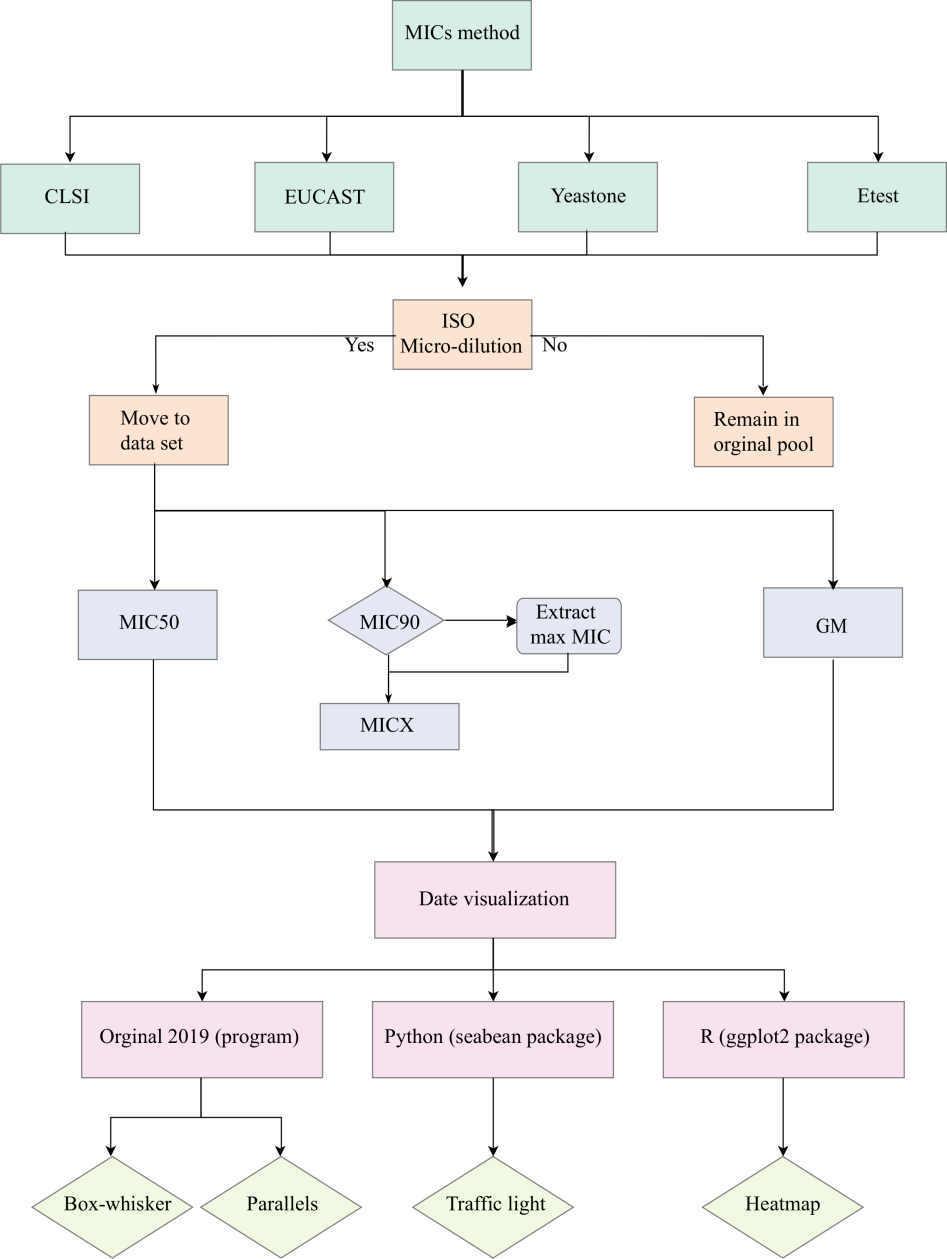
Flow chart of data collection and visualization.

A flowchart of applied methodology is given in Fig. 1. Data were extracted from the *Atlas of Clinical Fungi* (7), which includes full descriptions of 720 fungi published with proven infections in humans or other vertebrates. Over the entire kingdom Fungi (Eumycota), antifungal data are available for 34 orders covering 318 species (Table S1). Data are listed with species identity and phylogenetic information, antifungal agents, study year, location, number of isolates tested, test method, MIC values (MIC ranges, MIC_50_, 50% inhibition relative to untreated control, and MIC_90_, 90% inhibition relative to untreated control, and GM, geometric mean), and reference (35). The database covers more than 300 papers on antifungal susceptibility. For the current meta-analysis, four classes of antifungals were compared, that is, allylamines (terbinafine: TBF), azoles (fluconazole: FCZ, itraconazole: ITZ, posaconazole: PCZ, voriconazole: VCZ, ketoconazole: KTZ), echinocandins (caspofungin: CAS), and polyenes (amphotericin B: AMB) (Table 1), as on these eight compounds sufficient data have been published to allow comparison over the entire fungal kingdom (Table S1). Susceptibility test results with methods other than microdilution were discarded. For numerous species, MIC_90_ is close to the maximum number of MICs tested. For those fungi where MIC_90_ data were not available, the MAX of MICs was used as and renamed as MICX. The GM reflects the average trend in MIC values per taxonomic entity.

**TABLE 1:** Compared antifungals, with class affiliation and abbreviation

Class	Subclass	Antifungal agent	Abbreviation
Azoles	Imidazoles	Ketoconazole	KTZ
	Triazoles	Fluconazole, itraconazole, posaconazole, voriconazole	FCZ, ITZ, PCZ, VCZ
Polyenes		Amphotericin	AMB
Allylamines		Terbinafine	TBF
Echinocandins		Caspofungin	CAS

### Data visualization and analysis

#### Box–whisker plots

**Fig 2:**
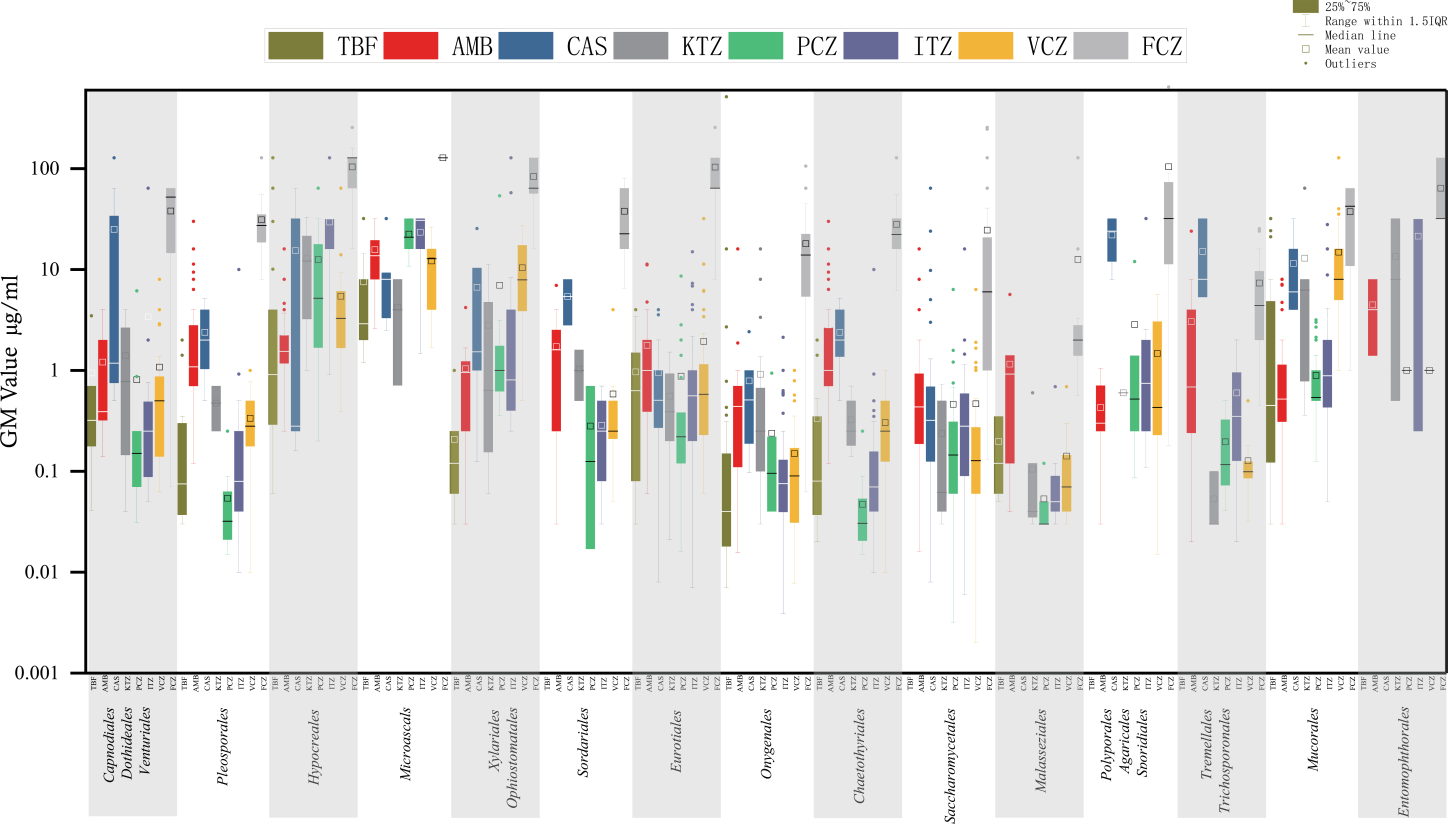
Box–whisker plot of GM values of the 15 ordinal groups of the fungi kingdom listed in Table 2. Eight antifungal agents are shown with colored labels, arranged at the X1-axis, in order of appearance: allylamine (TBF), polyene (AMB), echinocandin (CAS), long-tailed azoles (KTZ, PCZ, and ITZ), and short-tailed azoles (VCZ and FCZ).

Originlab 2019 software (OriginLab Corporation, Northampton, MA, USA) was used for box–whisker plots which were statistically analyzed separately in each order. The orders with insufficient data to construct separate plots were nevertheless included in the phylogenetic tree. Several small, phylogenetically related orders forming a monophyletic group in the tree were combined in a single plot, leading to a total of 15 groups (A-O; Table 2). GM values were used, remaining data MIC_50_, MICX are given in supplementary data (Fig. S1). The box length shows the 50% values ranging within 1.5 interquartile range, which means 99% of data are included, while outliers are shown as unique points. Median lines show average value levels and rectangles represent mean values. The antifungal agents were arranged at X1-axis, from start to end: allylamine (TBF), polyene (AMB), echinocandin (CAS), long-tailed azoles (KTZ, PCZ, and ITZ), and short-tailed azoles (VCZ and FCZ). Basic colors were used to distinguish the four classes, with color brightness indicating the individual compounds, leading to a total of eight colors. As FCZ is exclusively used for the treatment of yeast infections but not for filamentous fungal infections, only the orders *Agaricales, Polyporales, Saccharomycetales, Sporidiales, Tremellales*, and *Trichosporonales* show the FCZ color. X2-axis displays the order groups, and subsequently we generated separate MIC_50_ (Fig. S1), MICX (Fig. S2), and GM (Fig. 2) box–whisker plots. Accordingly, each line represents the summary of a species taken from the *Atlas of Clinical Fungi*. Abbreviations of antifungal names are according to *Atlas of Clinical Fungi* (7).

**TABLE 2:** Overview of orders treated in *Atlas,* and species allowing antifungal comparison

Order	*n* = Species in *Atlas*	*n* = Species in plot	Phylogenetic group show all^*a*^	Taxa containing opportunists (O) or animal/human-pathogenic fungi (P)
*Dothideales*	10	5	A	O
*Capnodiales*	6	3	A	O
*Venturiales*	4	4	A	O
*Pleosporales*	76	36	B	O
*Botryosphaeriales*	1	0	–	O
*Hypocreales*	48	30	C	O
*Microascales*	11	8	D	O
*Xylariales*	4	2	E	O
*Ophiostomatales*	11	5	E	O
*Diaporthales*	4	0	–	O
*Sordariales*	48	20	F	O
*Eurotiales*	99	42	G	O
*Arachnomycetales*	3	0	–	O
*Onygenales*	93	38	H	O/P
*Chaetothyriales*	58	34	I	O
*Saccharomycetales*	66	40	J	O
*Pneumocystidales*	2	0	–	O
*Wallemiales*	1	0	–	O
*Ustilaginales*	3	1	K	O
*Malasseziales*	16	8	K	O
*Entylomatales*	1	0	–	O
*Microstromatales*	1	0	–	O
*Sporidiales*	4	3	L	O
*Polyporales*	10	4	L	O
*Agaricales*	5	3	L	O
*Tremellales*	8	2	M	O
*Trichosporonales*	13	7	M	O
*Mucorales*	34	19	N	O
*Entomophthorales*	4	4	O	O
*Hymenochaetales*	4	0	–	O
*Mytilinidiales*	1	0	–	O
*Botryosphaeriales*	1	0	–	O
*Mortierellales*	2	0	–	O
*Chytridiales*	2	0	–	O

^*a*^ Classified by phylogenetic tree from top to bottom.

#### Parallel coordinate plots

**Fig 3:**
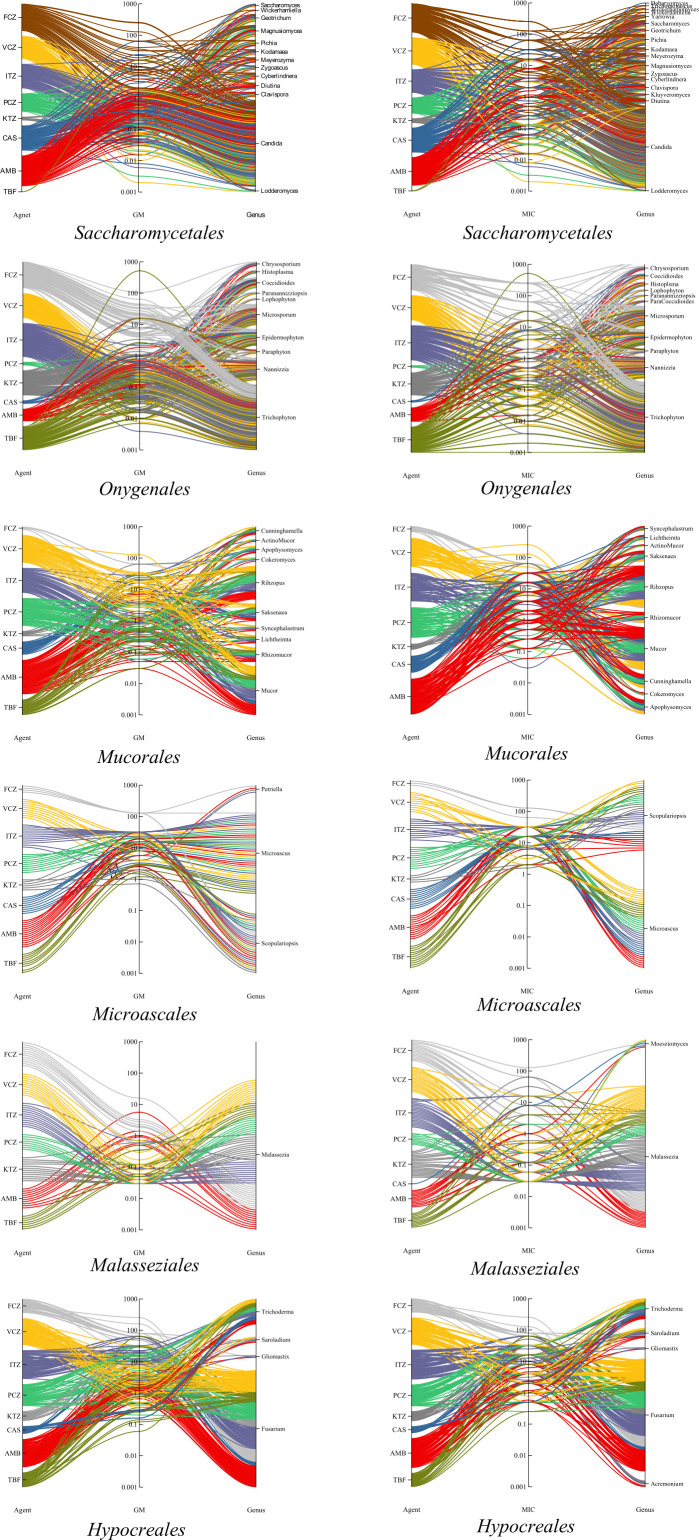
Parallel coordinate plot GM and MIC values of *Hypocreales*, *Microascales*, *Mucorales*, *Saccharomycetales*, and *Malasseziales*. MIC and GM values are plotted separately per antifungal and per species (generic names listed), with GM at the left and MIC at the right. Antifungal drugs arranged at left Y-axis, from top to bottom: short-tailed azoles (FCZ and VCZ), long-tailed azoles (ITZ, PCZ, and KTZ), echinocandins (CAS), and polyenes (AMB and TBF). Eight colors were used to distinguish the antifungals. As FCZ is applied in yeasts but not in filamentous fungi, only the orders *Agaricales*, *Polyporales*, *Saccharomycetales*, *Sporidiales*, *Tremellales*, and *Trichosporonales* show the FCZ color. Right Y-axis displays the genera; the lines connected with the antifungal via the central axis are summarized values of individual species. Left and right columns are connected for each species. Accordingly, each line represents the summary of a species taken from the *Atlas of Clinical Fungi*.

Data points are presented in parallel coordinate plots using Originlab 2019 software. In each plot, fungal species were classified according to ordinal affiliation (classification taken from www.indexfungorum.org) and separate plots were made for each order. We selected the orders *Mucorales*, *Microascales*, *Hypocreales*, *Malasseziales, Saccharomycetales*, and *Onygenales* to visualize differential trends between groups that are phylogenetically remote (Fig. 3); remaining orders are given in supplementary data (Fig. S4). MICX and GM values were plotted separately per antifungal and per genus. The antifungal drugs were arranged at left Y-axis corresponding to box–whisker plots X1-axis, with the lines connected with the antifungal via the central axis are summarized values of individual species in mentioned genera. Left and right columns are connected for each species, whereby the connecting line centrally touches the calculated MICX or GM value for that species. The different densities between orders are due to varying amounts of literature data.

#### Traffic light

**Fig 4:**
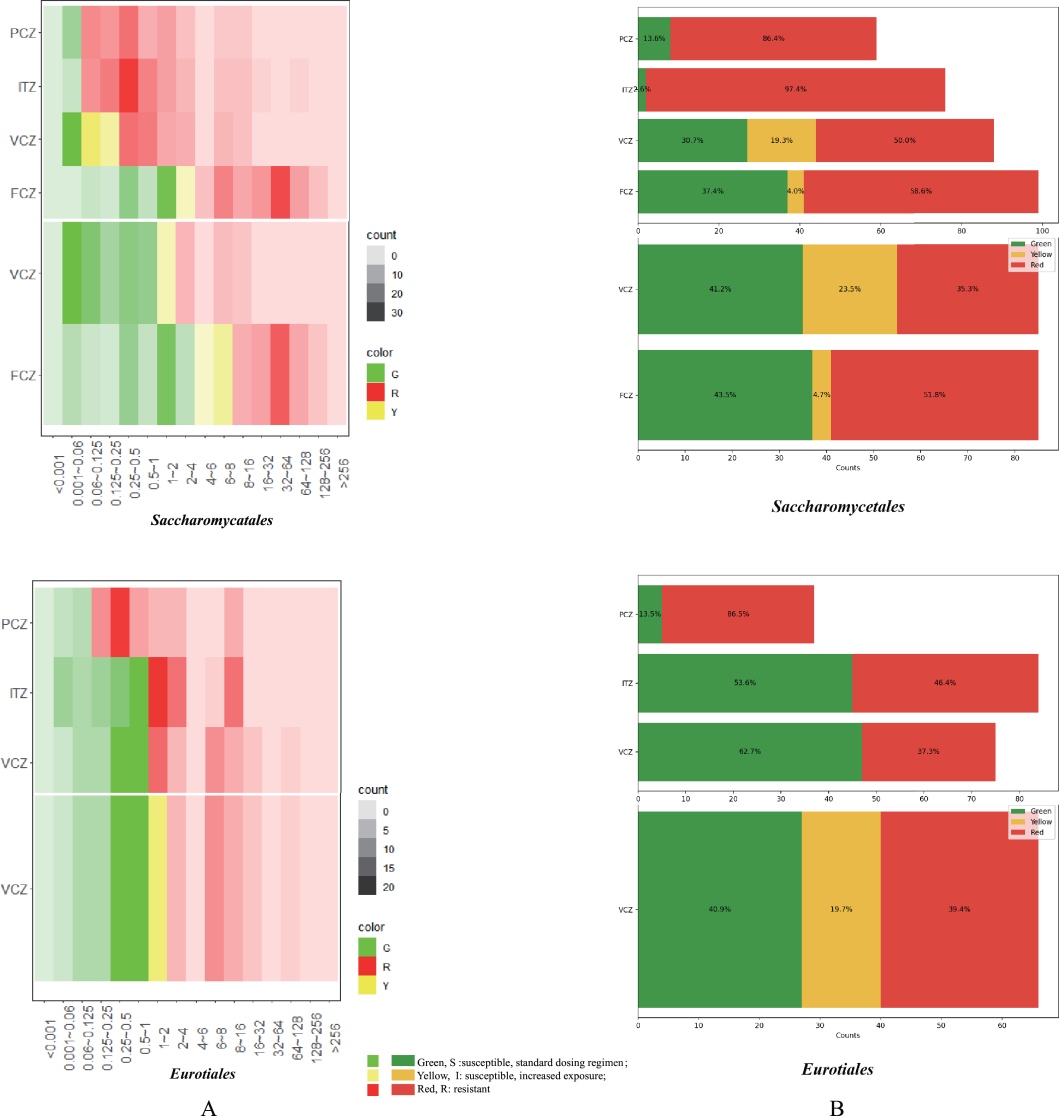
Two methods CBP were compared in the orders *Eurotiales* and the *Saccharomycetales* as an example. EUCAST CBP displayed at the top, CLSI CBP at the bottom. Fig. 4A is the heatmap result, Fig. 4B is the traffic light result.

Based on EUCAST CBPs for *Candida* and *Aspergillus* (v11.0, https://www.eucast.org/) (Table 3), Matploptil (36) was used to make stacked bar charts. *C. albicans* was considered representative for yeasts, and *A. fumigatus* for filamentous fungi; breakpoints are listed in Table 3. Data are listed for ITZ, PCZ, and VCZ, eventually supplemented with FCZ where sufficient data are available. MICX data in our database were used. The following EUCAST (https://www.eucast.org/newsiandr/) (version 2022) indications are used: *S* (susceptible with standard dosing regimen) shown in green, intermediate *I* (susceptible with increased exposure) shown in yellow, and *R* (resistant) shown in red. Examples are given of the most frequently reported orders, *Eurotiales* and *Saccharomycetales* (Fig. 4B). The X-axis (Fig. 4B; Fig. S5) represents the counts of species that are susceptible/intermediate/resistant per azole per order. Percentages of *S*, *I,* and *R* for each category in all studied orders are shown in Fig. S5. In Fig. S5, CLSI breakpoints for yeasts (M60, ed. 2; 37) and filamentous fungi (M61, ed. 2; 35) were also used. We selected the breakpoints of the same species *C. albicans* and *A. fumigatus* to compose CLSI breakpoint traffic lights for all orders.

**TABLE 3:** EUCAST clinical breakpoints (v11.0) for interpretation of MICs for antifungal agents^*a*^

Species	Agents	*S*	*I*	*R*
*Candida albicans*	FCZ	≤2	4	>4
	ITZ	≤0.06		>0.06
	PCZ	≤0.06		>0.06
	VCZ	≤0.06	0.125–0.25	>0.25
*Aspergillus fumigatus*	ITZ	≤1		>1
	PCZ	≤0.125		>0.25
	VCZ	≤1		>1

^*a*^ *S:* susceptible, standard dosing regimen; *I*: susceptible, increased exposure; *R*: resistant. https://www.eucast.org/clinical_breakpoints.

#### Heatmap

To show the distribution trends of MICs under different dilutions of antifungal agents (Fig. 4A), we used the same data and CBPs as the traffic light charts (Fig. 4B; Fig. S5). A script based on the R package, ggplot2 (38) was applied, with an X-axis corresponding to drug concentrations 0.001, 0.006, 0.06, 0.125, 0.25, 0.5, 1, 2, 4, 8, 16, 32, 64, 128, 256, and >256 mg/L. Susceptible *S* is shown in green, intermediate *I* in yellow, and resistant *R* in red. The color alpha indicates the data point counts for each box (Fig. S6). Results of *Eurotiales* and *Saccharomycetales,* both having large amounts of data points, are given as an example (Fig. 4B). CLSI breakpoints are shown according to the same flowchart as EUCAST (Fig. S6), this Figure also shows a comparison of the results.

### Phylogeny

**Fig 5:**
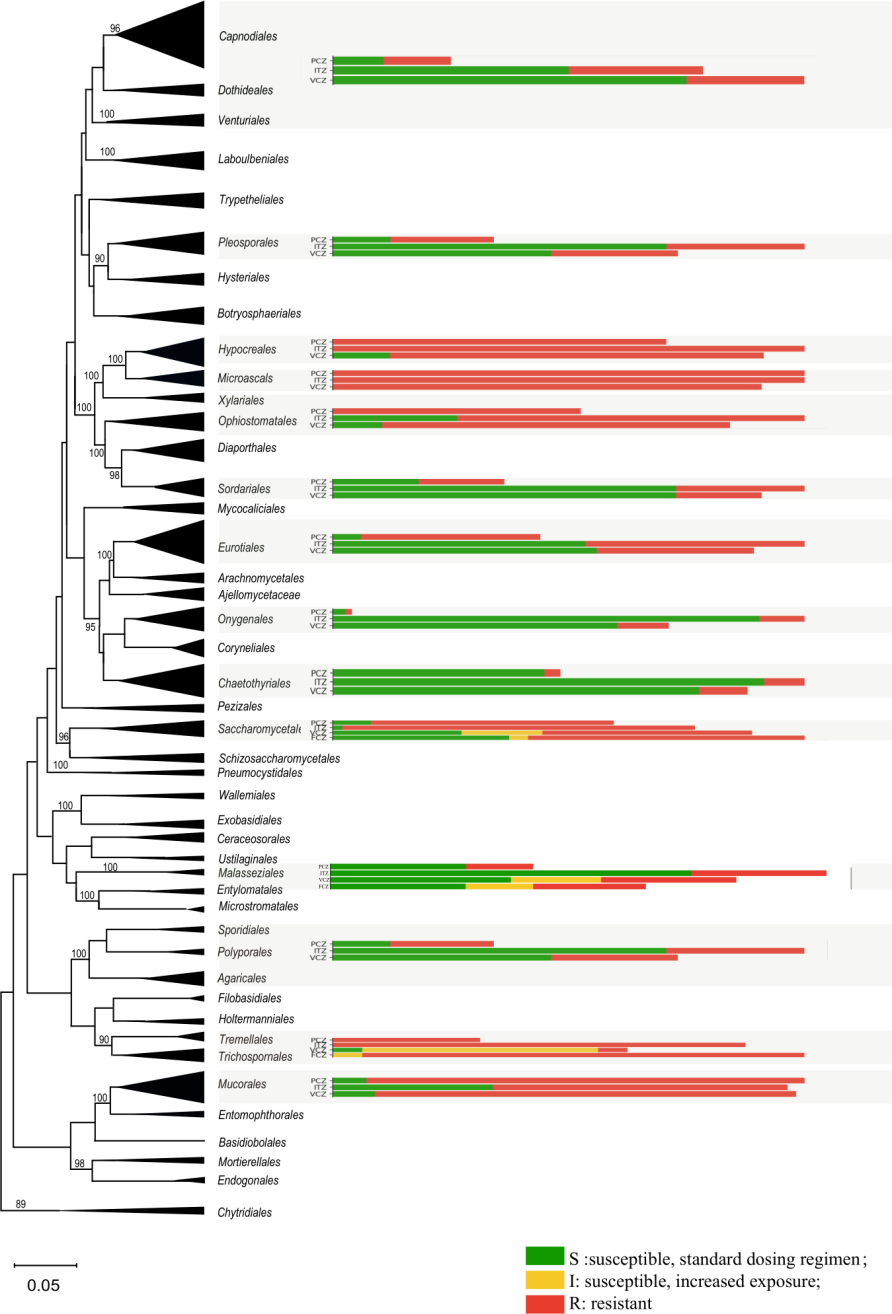
Maximum likelihood tree of the fungal kingdom combined with antifungal data in traffic light format. Thirty-four clinically relevant orders and 11 non-clinical orders are included. Red bars display >50% resistance, green bars >50% susceptible; yellow = intermediate.

The D1/D2 regions of large subunit rRNA (LSU) regions of representatives of 34 clinically relevant orders and 11 non-clinical orders sequences were downloaded from GenBank and aligned with MAFFT v7 (39). Sequences were analyzed using Maximum Likelihood (ML) algorithm in the CIPRES Science Gateway portal (www.phylo.org, accessed on 1 December 2021) (40). Maximum Likelihood analyses were run using RAxML v8.2.10 (41) with default parameters. The ML bootstraps were computed after 1,000 replicates. The resulting tree from ML analysis was edited in MEGA v11.0. Bootstrap values > 80% are shown (Fig. 5). Of selected orders, the available traffic light data of Fig. S5 are presented next to the collapsed ordinal cluster; the X-axis presents the data point counts.

## RESULTS

A recent overview (7) of antifungal susceptibility data in the kingdom fungi (Eumycota) provided susceptibility profiles of 318 species belonging to 34 orders (Table S1). Responses to eight antifungal agents (AMB, CAS, FCZ, ITZ, KTZ, PCZ, TBF, and VCZ) were included. Members of the orders *Entomophthorales, Hypocreales*, *Microascales, Mucorales*, the combined *Ophiostomatales* and *Xylariales*, and most basidiomycetes (orders *Agaricales/Polyporales/Sporidiales*, and *Tremellales/Trichosporonales*) all showed consistently high MIC values for almost all antifungal agents (Fig. S4C, G, E, and M). Consistent susceptibility is noted in the filamentous ascomycete orders *Chaetothyriales, Onygenales, Pleosporales, Sordariales*, the combined clade *Capnodiales/Dothideales/Venturiales*, and the basidiomycetous yeast order *Malasseziales*. The *Eurotiales* and *Saccharomycetales* take a somewhat intermediate position. FCZ is *in vitro* effective against ascomycetous yeasts (order *Saccharomycetales*) only. The compound has also widely been tested in the basidiomycetous yeast orders *Sporidiales, Tremellales*, and *Trichosporonales*, but rarely in remaining orders, where data on this compound are scant.

### Box–whisker

MIC_50_, MIC_90_, and GM data showed optimal differentiation between groups with the use of box–whisker plots (Fig. 2; Fig. S1 and S2). In all plots, exemplified by the display of GM values (Fig. 2), FCZ (gray) shows relatively high values, usually significantly higher than those of remaining antifungals in the same group and in nearly all groups outside the range of variability of other agents. In *Saccharomycetales*, absolute FCZ values are somewhat lower, though still consistently high compared to other agents in the same order. CAS (blue) yielded relatively high values in 6 of the 14 (groups of) orders, particularly in the basidiomycetes (*Agaricales, Polysporales, Sporidiales, Tremellales*, and *Trichosporonales*; for *Malasseziales* insufficient data were available). In addition, obtained values of the compound showed a large spread in three of the groups (*Capnodiales/Dothideales/Venturiales, Hypocreales*, and *Ophiostomatales/Xylariales*). *Entomophthorales, Hypocreales*, *Microascales*, and *Mucorales* showed consistently high GM, MIC values for almost all antifungal agents. In contrast, members of *Malasseziales, Ustilaginales*, and *Chaetothyriales* were relative sensitive to azoles other than FCZ. Similarly, *Saccharomycetales* were equally susceptible to all antifungals reviewed, while *Chaetothyriales* showed the largest intra-ordinal variation (Fig. 2). The largest difference in susceptibility to azoles vs AMB (red) and CAS (blue) was noted in *Tremellales/Trichosporonales*. Among the azoles, PCZ (green) mostly yielded rather consistent data which were similar within orders, whereas VCZ (ochre) showed larger ranges of variability, particularly in *Entomophthorales*. TBF values were mostly low, particularly in *Ophiostomatales/Xylariales* and *Malasseziales,* while the largest ranges of variability were noted in the multi-resistant orders *Microascales* and *Mucorales* (Fig. 2).

In the box–whisker plot using MIC_90_ as input data (Fig. S1), the poor performance of FCZ is less conspicuous. The orders *Pleosporales* and *Mucorales* show average susceptibility for this compound, while CAS showed higher resistance. In *Chaetothyiales*, *Trichosporonales*, and *Tremellales* all azoles except FCZ yield relatively low values. VCZ, ITZ, KTZ, and PCZ demonstrated large degrees of variability between species. In the MIC_50_ plot (Fig. S2), the spread is more pronounced, being available in a larger number of species. *Sordariales* perform similarly as *Malasseziales* and *Ustilaginales* in azole susceptibility, being relatively susceptible to agents other than FCZ. TBF shows low values in *Xylariales*, *Ophiostomatales*, *Capnodiales*, *Dothideales*, and *Venturiales* (Fig. S2).

Significant differences are observed between three main groups of Ascomycota frequently involved in systemic infections, that is, ascomycetous yeasts (*Saccharomycetales* comprising *Candida* spp.) and *Aspergillus* (member of *Eurotiale*s) on the one hand, with the dimorphic fungi (*Blastomyces, Emergomyces*, *Histoplasma*, and relatives in *Onygenales*) on the other (Fig. S3). The dimorphic fungi show relatively consistent profiles, with little spread and no outliers. FCZ again shows the highest degree of resistance of tested antifungals, particularly in dimorphic fungi. *Onygenales* on average are more sensitive to five remaining drugs (for CAS, KTZ, and TBF insufficient data were available), with low medians and GMs. Between *Aspergillus* and *Candida*, differences are smaller, although on average the yeasts are slightly more susceptible to azole agents than *Aspergillus*. For TBF, this trend seems to be the opposite, but this result may have been impacted by limited availability of data in *Saccharomycetales*.

### Parallel coordinate

These plots show the values of individual species. MIC_90_ data are used to judge the degree of antifungal resistance, while GM is important to compare the effectivity of antifungal agents between species. The latter is a more accurate measure of the general tendency within the species, mitigating the effect of the extremes. Therefore, all plots are shown in double, whereby MIC_90_ values are characterized by discrete intervals, and GM data are continuous. In *Microascales*, high MIC_90_ values are found for all agents. In members of this order, CAS, FCZ, and VCZ consistently show high MIC_90_ values, which is also reflected in the GM (Fig. S4). In the order *Entomophthorales*, the high MIC_90_ values were consistent with GM and MIC_50_ for FCZ, AMB, KTZ, and ITZ. In *Basidiobolus*, MICs are somewhat lower than those of *Conidiobolus* (Fig. S4).

Antifungal resistance in *Hypocreales* is on average slightly lower than that in *Microascales*. This is demonstrated by *Fusarium*, the largest genus in *Hypocreales* (Fig. 3), showing a wide range of responses to AMB and TBF; most species had high MIC_90_ values, but lower values also occurred. Other hypocrealean genera, that is, *Acremonium, Gliomastix*, and *Trichoderma* shared high MIC_90_ values for all agents analyzed. However, while GM values in these genera for azoles were all at a high level, AMB and TBF ranged in the same species between high and low. All species of the orders *Malasseziales, Chaetothyriales*, and *Onygenales* demonstrated pronounced susceptibility to ITZ, PCZ, KTZ, and VCZ (Fig. 2; Fig. S4), with very few deviating strains.

### Traffic light

The traffic light plot (Fig. S5) shows the number of susceptible vs resistant species according to EUCAST breakpoint recommendations for *C. albicans* and *A. fumigatus*. Only for *Candida*, the intermediate category *I* was introduced, while for filamentous fungi the values are interpreted as either *S* or *R*. For ascomycetous and basidiomycetous yeasts, sufficient FCZ data were available, but for filamentous fungi, only three compounds (ITZ, PCZ, and VCZ) could be compared. Over the entire fungal kingdom, data representing 22 orders were collected, leading to 14 traffic light graphics (Fig. S5). The X-axis of the graphics denote the number of species analyzed. Extrapolating the EUCAST values to other fungi, a large number of orders displays significant resistance. The most extreme order is *Microascales*, where no susceptibility is observed. The most susceptible orders are *Malasseziales* (lipophilic skin colonizers) and *Chaetothyriales* (black yeasts and allies). In summary, by using *C. albicans* and *A. fumigatus* EUCAST breakpoints as standard, the following orders can be listed as being commonly susceptible: *Capnodiales/Dothideales/Venturiales, Malasseziales, Onygenales, Pleosporales*, and *Sordariales*, while resistance is prevalent in *Agaricales/Polyporales/Sporidiales, Hypocreales, Microascales, Mucorales, Ophiostomatales/Xylariales*, and *Tremellales/Trichosporonales*. The orders *Eurotiales* (containing *Aspergillus*) and *Saccharomycetales* (containing *Candida*) take a somewhat intermediate position.

All orders of basidiomycetous yeasts and filamentous basidiomycetes analyzed (*Agaricales, Polyporales, Sporidiales, Tremellales*, and *Trichosporonales*) showed 100% resistance to the long-tailed azoles ITZ and PCZ. In contrast, sensitivities against these compounds in ascomycetous yeasts (*Saccharomycetales*) ranged between 30.7% and 37.4%. This order also showed more susceptibility to FCZ and VCZ than the basidiomycetous members of *Sporidiales/Polyporales/Agaricales*, which demonstrated 9.1%–12.5% sensitivity. In general, significant antifungal resistance is apparent in Basidiomycota, except for 80% intermediate resistance to VCZ in *Tremellales/Trichosporonales*.

The intrinsically resistant orders *Hypocreales* and *Microascales* are among the fungi with highest degrees of multi-resistance. The long-tailed azole ITZ yielded 35.2% and PCZ 7.1% susceptibility; 9.1% was susceptible to the short-tailed azole VCZ. *Ophiostomatales/Xylariales* were 100% resistant to PCZ, 87.5% to VCZ, and 73.7% to ITZ. This is significantly different from the most susceptible orders, *Malasseziales* (ITZ 9.1%, PCZ 12.5%, and VCZ 5.3%) and *Chaetothyriales* (ITZ 8.6%, PCZ 7.1%, and VCZ 11.8%). In the order *Onygenales*, averaged over dermatophytes and dimorphic pathogens, the values for ITZ and VCZ were 9.6% and 15.4%, respectively. The uncommon environmental opportunists in *Capnodiales, Dothideales Pleosporales, Sordariales*, and *Venturiales* all showed more than 50% counts in the susceptible range to VCZ and ITZ, but in these orders around 50% resistance to PCZ is observed. High degrees of sensitivity to PCZ, ITZ, and VCZ are limited to the two most susceptible orders, *Chaetothyriales* and *Malasseziales*.

A breakpoint traffic light plot based on CLSI criteria (Fig. S5) showed similar trends in the orders investigated. The intrinsically resistant orders *Hypocreales* (88.5%), *Microascales* (100%), and *Mucorales* (90.9%) yielded high percentages of resistance to VCZ, followed by *Ophiostomatales/Xylariales* (87.5%). The orders *Tremellales/Trichosporonales* (87.5%) were more resistant than other yeast orders. Resistance to FCZ was invariably high outside *Saccharomycetales*.

Comparing our quantifications based on CBP criteria of EUCAST vs CLSI (Fig. S5), percentages of resistance in orders of filamentous fungi proved closely similar. In the CLSI system, 1 mg/µL is listed as *I*, but EUCAST did not have *I* in *A. fumigatus* CBP. Consequently, the sum of the percentages of *S* and *I* according to CLSI is equal to *S* according to EUCAST criteria. For the yeast orders, the resistance percentage decreases and the susceptibility percentage increases, except for *Sporidiales/Polyporales/Agaricales*.

### Heatmaps

The heatmap summary shows the distribution of data points of MICs in the analyzed orders, providing a different angle demonstrating *S*, *I*, and *R* based on EUCAST criteria (Fig. S6). Concerning resistance against the long-tailed azoles PCZ and ITZ, MIC values of the intrinsically resistant filamentous orders *Hypocreales* and *Microascales* were found concentrated in the higher ranges. In *Mucorales,* values against PCZ were concentrated in lower ranges, while the resistance distribution against ITZ was scattered, and that against VCZ was concentrated in higher ranges. In the intrinsically resistant orders *Ophiostomatales/Xylariales*, values were concentrated in the higher ranges of the resistance interval of VCZ, but scattered with the long-tailed azoles PCZ and ITZ. Of the basidiomycetous yeasts *Tremellales/Trichosporonales*, and *Sporidiales/Polyporales/Agaricales*, showing relatively high resistance ratios, only ITZ values of *Tremellales/Trichosporonales* were concentrated in lower ranges, and the remaining values in both PCZ and ITZ were scattered. The short-tailed azoles VCZ and FCZ yielded values in higher ranges showing with *Sporidiales/Polyporales/Agaricales*, while those of remaining basidiomycetes showed relatively uniform distribution over a wide range. Among the relatively susceptible filamentous orders, MIC values of *Eurotiales* and *Pleosporales* were relatively close to *R* in ITZ and VCZ. The highly susceptible orders/order groups *Chaetothyriales* and *Capnodiales/Dothideales/Venturiales* were concentrated in lower ranges, while values in *Onygenales* and *Sordariales* were more dispersed. Two methods for CBP determination focused on *Eurotiales* and *Saccharomycetales* are shown in Fig. 4.

### Phylogenetic summary

Subsequently, we plotted the EUCAST traffic light charts (Fig. S5) on an LSU phylogenetic tree of the fungal kingdom, based on 34 orders containing clinical representatives and 11 orders of strictly non-clinical fungi; the total selection was biased toward taxa containing members potential able to cause human infection (Fig. 5). The upper part of the tree contains the filamentous Ascomycota, allowing distinction of three classes: *Dothidiomycetes* (containing orders *Capnodiales/Dothideales/Venturiales* and *Pleosporale*s included in the study), *Sordariomycetes* (containing orders *Hypocreales, Microascales*, and *Ophiostomatales/Xylariales* and *Sordariales*) and *Eurotiomycetes* (containing orders *Chaetothyriales, Eurotiales*, and *Onygenales*). In the lower part of the tree, ancestral groups show large distances between clades. In the present tree, two approximate class-level groups can be recognized, attributable as *Ustilaginomycetes* (smut-like fungi: order *Malasseziales*) and *Tremellomycetes* (heterobasidiomycetes, containing orders *Agaricales, Polyporales*, and *Sporidiales*). In the lower fungi, the *Mucorales* and *Entomophothorales* form a clade, albeit at very large distance.

In the LSU phylogeny of the fungal kingdom, the most significant resistance is observed in the *Sordariomycetes*, with the orders *Microascales, Hypocreales*, *Ophiostomatales/Xylariales*, and *Sordariales*. The sister-branches of *Hypocreales* and *Microascales* share the highest MIC values. High values against FCZ are found over the entire fungal kingdom, including in most species of *Saccharomycetales*. In the ascomycetous yeasts (*Saccharomycetales*) average resistance is observed against most other compounds. Pronounced susceptibility is observed in *Eurotiomycetes* with the low to average resistant orders *Chaetothyriales, Eurotiales*, and *Onygenales*. Similar data are observed in the large class *Dothideomycetes*, but since this group comprises very few clinically relevant species, data are insufficient for a reliable comparison. High degrees of resistance are noted in most basidiomycete class *Tremellomycetes*, while the opposite is observed in the *Malasseziales* which are phylogenetically affiliated to the smut-like fungi. The lower fungi also show high degrees of resistance.

## DISCUSSION

Among fungal opportunists, a large variation in degrees of antifungal susceptibility is known to exist. Therefore, appropriate clinical management carefully considers the antifungal susceptibility profile of the etiologic agent against antifungal drugs. Related fungi often show comparable trends in resistance or susceptibility. McGinnis and Pasarell were the first to investigate this systematically by comparing antifungal profiles with the position of the analyzed fungi in the phylogenetic system (42). Today, more data are available, both in molecular phylogeny and in antifungal susceptibility test (AFST) results, and this allows a more thorough analysis of the behavior of divergent opportunists toward various classes of antifungal agents.

The *Atlas of Clinical Fungi* (7) summarizes a large amount of published data on antifungal susceptibility of pathogenic and opportunistic fungi. In the literature, values of sensitivity, recorded as MICs, have variously been presented as MIC ranges, MIC_50_, MIC_90_, GM, or as epidemiological cut-off values (ECVs or ECOFFs). This diversity interferes with meta-analyses of published data. MIC_90_ or MIC combined with recognized CBPs are preferably used to guide clinical treatment. Breakpoints determine two essential values of drug interactions, that is, resistance vs susceptibility, eventually with an intermediate zone. Advisably, higher drug levels than the breakpoints are used in clinical practice in order to reliably obtain successful treatment. Determination of phenotypic susceptibility profiles per species allows establishment of a cut-off value marking the borderline between *in vitro* susceptibility and resistance, known as ECV or ECOFF or wild-type cut-off value. Species affiliation does not always predict levels of sensitivity, as individual strains may have acquired resistance to the drug(s) to which the wild-type population is sensitive. For fungi with acquired resistance, the ECV is different from the breakpoint value. In clinical settings, the values assist in selecting the most likely effective antifungal, although patients may respond well despite determined fungal *in vitro* resistance to the drug.

Standardized for AFST of yeast (M27-A2) and filamentous fungi (M38-A) have been published by CLSI and EUCAST. With the emergence of novel antifungal agents, such as the new generations of triazoles and echinocandins, protocols have been expanded and improved. However, this is a complicated and time-consuming process. We reviewed published and *in vitro* antifungal data generated following CLSI and EUCAST protocols, which demonstrate some technical differences but are sufficiently harmonized to allow meaningful comparison over larger phylogenetic groups. Both standards were derived from the microdilution method, and adjustments for species-specific and more sensitive CBP suggest that FCZ and VCZ are rather similar, and that both methods can be used to identify and monitor responses *in vitro*. As a consequence, also ECVs and breakpoints deviate between both standards. Both methods can distinguish wild-type strains and resistant strains, and between the two methods MICs can differ by as much as three dilutions. EUCAST and the more pragmatic CLSI 24 hours protocols of triazoles, AMB, and flucytosine produced comparable MIC results (32, 43–45).

Considering the above, we combined all data generated by either one of the two protocols for determining CBPs according to EUCAST and CLSI, in order to show trends of resistance in the fungal kingdom. At this meta-level, the overall results are consistent. The differences in breakpoint determination affect the distribution gradient of resistance vs susceptibility, which in turn affects the percentage of drug resistance. EUCAST includes more species with antifungal breakpoints than CLSI, we therefore chose the breakpoints of EUCAST to compare levels of susceptibility over larger phylogenetic groups in the fungal kingdom, that is, at the level of orders, or sometimes combinations of orders.

Despite intra- and interspecies variation in antifungal resistance, significant consistency within and difference between phylogenetic groups were observed. Large sections of the fungal kingdom represented “resistance” on the basis of EUCAST criteria for yeasts and filamentous fungi (Fig. 5). In contrast to acquired resistance, this natural or intrinsic resistance demonstrates a common factor shared by an entire taxonomic/phylogenetic group, and is not an adaptation to a particular stress factor. The limited research done to date indicates that the mechanisms of acquired and intrinsic resistance might be similar (22) and supposedly do not have negative impact on the fitness of the species in the natural environment. We combined a simplified phylogenetic tree of the fungal kingdom with extrapolated breakpoint data from 318 species collected in the *Atlas of Clinical Fungi* (7). Somewhat contrary to expectations, large parts of the fungal kingdom showed intrinsic MDR. Particularly the *Sordariomycetes*, with the almost fully resistant sister orders *Hypocreales* and *Microascales*, show preponderance of natural resistance. Although difficult to generalize for the thousands of members of this class, the group shows an ecological association with dung, agricultural soil, polluted habitats, and opportunism on a variety of weakened plant species, suggesting eutrophy. Significant resistance is also observed in *Mucorales* and *Entomophthorales* (sister branches in Fig. 5, but at considerable distance), orders which contain fungi without nutritional specialization and found in rich substrates in early stages of decay, including intestines of lower animals. High degrees of resistance are also found in the basidiomycetous clade. This group has been split up in a large number of subclasses because of large phylogenetic distances, and levels of antifungal resistance differ markedly between orders. The orders *Agaricales, Polyporales, Tremellales*, and *Trichosporonales* have wood decay or hyperparasitism on other fungi as important ecological factors, and high degrees of resistance. In contrast, the *Malasseziales*, a small order of lipid-colonizers of mammal skin and fur, is highly susceptible. In the filamentous ascomycetes, high degrees of sensitivity are found in the subclass *Eurotiomycetes* containing orders *Chaetothyriales, Eurotiales*, and *Onygenales*, the last-mentioned order comprising the dermatophytes and dimorphic pathogens. The *Chaetothyriales*, comprising black yeasts and relatives with xenobiotic assimilation abilities, are mostly *in vitro* susceptible despite clinical recalcitrance (46). Remarkably, the two orders that are widely used to standardize antifungal parameters, that is, the *Eurotiales* and *Saccharomycetales* containing *Aspergillus* and *Candida*, respectively, are actually somewhat exceptional in the fungal kingdom in their intermediate degrees of antifungal resistance (Fig. 5).

Intrinsic resistance is difficult to describe using current criteria, since the wild type is resistant and there is no dual distribution in resistance data. *Lomentospora prolificans* (order *Microascales*) is consistently resistant against several classes of antifungal drugs (47); in this fungus and its relatives, susceptible populations are lacking. In such fungi, breakpoint and ECV are identical. Intrinsic or natural resistance is opposed to acquired resistance, where susceptible populations prevail in the environment. Intrinsic resistance is found naturally among fungi without prior exposure to the drug. Since all strains of the species behave similarly by being resistant, the species affiliation is—apart from host factors—predictive for treatment success and this emphasizes the importance of identification of the etiologic agent. Resistance may concern a single compound, but more often multi-resistance is observed against different antifungal classes. The phenomenon has mostly been described *ad hoc* in particular species, such as in *C. krusei*, *C. auris*, and *N. albida* (synonym *C. albidus*). However, several entire genera and orders may show intrinsic MDR. Examples are the relationship of *Acremonium–Fusarium–Trichoderma* belonging to the order *Hypocreales*, and that of *Scedosporium–Lomentospora–Microascus–Scopulariopsis* belonging to the *Microascales*. Where in most fungi long-term exposure to antifungal drugs may lead to acquired resistance which is thought to be evolutionarily expensive (adaptive mutations, up-regulation of efflux pumps, obtaining foreign DNA, and other mechanisms) (48), intrinsic MDR species must have thrived since geological times with these conditions in absence of antifungals, thus without an apparent anthropogenic reason. In azole-polluted agricultural environments, both intrinsically azole-resistant species and strains with acquired azole resistance have a competitive advantage. Among the successful naturally resistant fungi are some common pest agents, such as *Batrachochytrium dendrobatidis* causing devastating epidemics in frog populations and driving ≈200 frog species close to extinction (49). The species is intrinsically short-tailed azole-resistant and was probably favored in fungicide-contaminated fresh water bodies (50, 51). Outbreaks or pseudo-epidemics of naturally multi-resistant species are also problematic in medical settings, where currently applied antifungal agents yield limited effects, as is obvious in the high case fatality rates of disseminated *Fusarium* infection (52–54) or by *C. auris* (21, 55).

Between resistance and susceptibility is a grey buffer zone known as intermediate response, which has however only been determined for two model species (Fig. S56). Awareness of this zone may prevent interpretive errors which can be circumvented by prescribing higher doses of the antifungal to achieve safe efficacy. As yet, precise definition of CBPs is limited to two model fungi, which is of great value for the majority of fungal infections, but are poorly representative for the hundreds of remaining opportunistic fungi. The present meta-analysis reveals a wide occurrence of fungi that are poorly susceptible to the currently used panel of antifungals. Among the relatively susceptible filamentous orders, MIC values of *Eurotiales* and *Pleosporales* were concentrated in the critical *S* and *R* intervals of ITZ and VCZ (Fig. S6). Although *in vitro* resistance does not exclude therapeutic success, the fact that hundreds of clinically relevant species are much less susceptible than the general reference of CBPs is worrying.

In this paper, we used four different types of presentations of the same data, to reliably convert the values gained for individual species to trends over the entire fungal kingdom. Parallel coordinate plots clearly showed the contributions of single data points to trends at ordinal levels and the number of data points in each order. Box–whisker plots deviate from other representations in showing the key values, such as average, median 25th percentile, and outliers are displayed; further, distributional features are shown such as whether the data are symmetrical, how tight the data are grouped, whether data are skewed and if so, in which direction. Focusing on azole MIC values, the heatmap provides the number of counts in different dilution steps. In order to overview the percentages of resistance in each group on the CBP scale, the traffic light graphic provides the optimal display of ordinal trends.

The present overview of antifungal profiles in the fungal kingdom is hampered by decreased comparability due to variable testing methods applied, and in addition, only half the number of extant opportunist are covered (7). Nevertheless this phylogenetic representation of AFST results demonstrate that current breakpoint data are poorly representative for numerous species outside the model taxa of breakpoint determination. Given the ever-expanding number of clinically relevant fungi, extended studies are overdue.

## SUPPLEMENTAL MATERIAL

Supplemental material spectrum.02127-23-s0001.pdf Figures S1 to S6.

ASM does not own the copyrights to Supplemental Material that may be linked to, or accessed through, an article. The authors have granted ASM a non-exclusive, world-wide license to publish the Supplemental Material files. Please contact the corresponding author directly for reuse.

## References

[1] Musial CE, Cockerill FR, Roberts GD. 1988. Fungal infections of the immunocompromised host: clinical and laboratory aspects. Clin Microbiol Rev 1:349–364. doi:10.1128/CMR.1.4.349 3069198 PMC358059

[2] Geerlings SE, Hoepelman AIM. 1999. Immune dysfunction in patients with diabetes mellitus (DM). FEMS Immunol Med Microbiol 26:259–265. doi:10.1111/j.1574-695X.1999.tb01397.x 10575137

[3] Mahfouz T, Anaissie E. 2003. Prevention of fungal infections in the immunocompromised host. Curr Opin Investig Drugs 4:974–990. 14508882

[4] Martino R, Salavert M, Parody R, Tomás JF, de la Cámara R, Vázquez L, Jarque I, Prieto E, Sastre JL, Gadea I, Pemán J, Sierra J. 2004. Blastoschizomyces capitatus infection in patients with leukemia: report of 26 cases. Clin Infect Dis 38:335–341. doi:10.1086/380643 14727202

[5] Rahi MS, Jindal V, Pednekar P, Parekh J, Gunasekaran K, Sharma S, Stender M, Jaiyesimi IA. 2021. Fungal infections in hematopoietic stem-cell transplant patients: a review of epidemiology, diagnosis, and management. Ther Adv Infect Dis 8:20499361211039050. doi:10.1177/20499361211039050 34434551 PMC8381463

[6] Bongomin F, Gago S, Oladele RO, Denning DW. 2017. Global and multi-national prevalence of fungal diseases—estimate precision. J Fungi (Basel) 3:57. doi:10.3390/jof3040057 29371573 PMC5753159

[7] de Hoog GS, de Guarro J, Gené JG, Ahmed SA, Al Hatmi AMS, Figueras MJ, Vitale RG. 2021. Atlas of clinical fungi: the ultimate benchtool for diagnostics. introductions, lower fungi, basidiomycetes, yeasts, filamentous ascomycetes AB. part Α. Foundation Atlas of Clinical Fungi.

[8] Vicente VA, Attili-Angelis D, Pie MR, Queiroz-Telles F, Cruz LM, Najafzadeh MJ, de Hoog GS, Zhao J, Pizzirani-Kleiner A. 2008. Environmental isolation of black yeast-like fungi involved in human infection. Stud Mycol 61:137–144. doi:10.3114/sim.2008.61.14 19287536 PMC2610314

[9] de S Araújo GR, Souza W de, Frases S. 2017. The hidden pathogenic potential of environmental fungi. Future Microbiol 12:1533–1540. doi:10.2217/fmb-2017-0124 29168657

[10] Denham ST, Wambaugh MA, Brown JCS. 2019. How environmental fungi cause a range of clinical outcomes in susceptible hosts. J Mol Biol 431:2982–3009. doi:10.1016/j.jmb.2019.05.003 31078554 PMC6646061

[11] van de Veerdonk FL, Gresnigt MS, Romani L, Netea MG, Latgé J-P. 2017. Aspergillus fumigatus morphology and dynamic host interactions. Nat Rev Microbiol 15:661–674. doi:10.1038/nrmicro.2017.90 28919635

[12] Hayes GE, Novak-Frazer L. 2016. Chronic pulmonary aspergillosis — where are we? and where are we going? J Fungi (Basel) 2:18. doi:10.3390/jof2020018 29376935 PMC5753080

[13] Spellberg B, Edwards J, Ibrahim A. 2005. Novel perspectives on mucormycosis: pathophysiology, presentation, and management. Clin Microbiol Rev 18:556–569. doi:10.1128/CMR.18.3.556-569.2005 16020690 PMC1195964

[14] Duggal P, Wise SK. 2013. Invasive fungal rhinosinusitis. Am J Rhinol Allergy 27:S28–S30. doi:10.2500/ajra.2013.27.3892 23711036

[15] Parikh SL, Venkatraman G, DelGaudio JM. 2004. Invasive fungal sinusitis: a 15-year review from a single institution. Am J Rhinol 18:75–81. 15152871

[16] Shi M, Mody CH. 2016. Fungal infection in the brain: What we learned from intravital imaging. Front Immunol 7:292. doi:10.3389/fimmu.2016.00292 27532000 PMC4969284

[17] Rawson TM, Moore LSP, Zhu N, Ranganathan N, Skolimowska K, Gilchrist M, Satta G, Cooke G, Holmes A. 2020. Bacterial and fungal coinfection in individuals with coronavirus: a rapid review to support COVID-19 antimicrobial prescribing. Clin Infect Dis 71:2459–2468. doi:10.1093/cid/ciaa530 32358954 PMC7197596

[18] Song G, Liang G, Liu W. 2020. Fungal co-infections associated with global COVID-19 pandemic: a clinical and diagnostic perspective from China. Mycopathologia 185:599–606. doi:10.1007/s11046-020-00462-9 32737747 PMC7394275

[19] Pemán J, Ruiz-Gaitán A, García-Vidal C, Salavert M, Ramírez P, Puchades F, García-Hita M, Alastruey-Izquierdo A, Quindós G. 2020. Fungal co-infection in COVID-19 patients: should we be concerned? Rev Iberoam Micol 37:41–46. doi:10.1016/j.riam.2020.07.001 33041191 PMC7489924

[20] Kontoyiannis DP. 2017. Antifungal resistance: an emerging reality and a global challenge. J Infect Dis 216:S431–S435. doi:10.1093/infdis/jix179 28911044

[21] Satoh K, Makimura K, Hasumi Y, Nishiyama Y, Uchida K, Yamaguchi H. 2009. Candida auris sp. nov., a novel ascomycetous yeast isolated from the external ear canal of an inpatient in a Japanese hospital. Microbiol Immunol 53:41–44. doi:10.1111/j.1348-0421.2008.00083.x 19161556

[22] Katiyar SK, Edlind TD. 2009. Role for Fks1 in the intrinsic echinocandin resistance of Fusarium solani as evidenced by hybrid expression in Saccharomyces cerevisiae. Antimicrob Agents Chemother 53:1772–1778. doi:10.1128/AAC.00020-09 19258277 PMC2681557

[23] Schoustra SE, Debets AJM, Rijs A, Zhang J, Snelders E, Leendertse PC, Melchers WJG, Rietveld AG, Zwaan BJ, Verweij PE. 2019. Environmental hotspots for azole resistance selection of Aspergillus fumigatus, the Netherlands. Emerg Infect Dis 25:1347–1353. doi:10.3201/eid2507.181625 31211684 PMC6590754

[24] Gisi U. 2022. Crossover between the control of fungal pathogens in medicine and the wider environment, and the threat of antifungal resistance. Plant Pathol 71:131–149. doi:10.1111/ppa.13429

[25] Myung K, Klittich CJR. 2015. Can agricultural fungicides accelerate the discovery of human antifungal drugs? Drug Discov Today 20:7–10. doi:10.1016/j.drudis.2014.08.010 25172802

[26] Jampilek J. 2016. Potential of agricultural fungicides for antifungal drug discovery. Expert Opin Drug Discov 11:1–9. doi:10.1517/17460441.2016.1110142 26549424

[27] Agnello S, Brand M, Chellat MF, Gazzola S, Riedl R. 2019. A structural view on medicinal chemistry strategies against drug resistance. Angew Chem Int Ed Engl 58:3300–3345. doi:10.1002/anie.201802416 29846032

[28] Jørgensen LN, Heick TM. 2021. Azole use in agriculture, horticulture, and wood preservation – is it indispensable? Front Cell Infect Microbiol 11:730297. doi:10.3389/fcimb.2021.730297 34557427 PMC8453013

[29] Verweij PE, Snelders E, Kema GHJ, Mellado E, Melchers WJG. 2009. Azole resistance in Aspergillus fumigatus: a side-effect of environmental fungicide use? Lancet Infect Dis 9:789–795. doi:10.1016/S1473-3099(09)70265-8 19926038

[30] Azevedo M-M, Faria-Ramos I, Cruz LC, Pina-Vaz C, Rodrigues AG. 2015. Genesis of azole antifungal resistance from agriculture to clinical settings. J Agric Food Chem 63:7463–7468. doi:10.1021/acs.jafc.5b02728 26289797

[31] Alcazar-Fuoli L, Mellado E. 2014. Current status of antifungal resistance and its impact on clinical practice. Br J Haematol 166:471–484. doi:10.1111/bjh.12896 24749533

[32] Albataineh MT, Sutton DA, Fothergill AW, Wiederhold NP. 2016. Update from the laboratory: clinical identification and susceptibility testing of fungi and trends in antifungal resistance. Infect Dis Clin North Am 30:13–35. doi:10.1016/j.idc.2015.10.014 26739605

[33] Pfaller MA, Diekema DJ, Ostrosky-Zeichner L, Rex JH, Alexander BD, Andes D, Brown SD, Chaturvedi V, Ghannoum MA, Knapp CC, Sheehan DJ, Walsh TJ. 2008. Correlation of MIC with outcome for Candida species tested against caspofungin, anidulafungin, and micafungin: analysis and proposal for interpretive MIC breakpoints. J Clin Microbiol 46:2620–2629. doi:10.1128/JCM.00566-08 18579718 PMC2519503

[34] Wayne P. 2002. Reference method for broth dilution antifungal susceptibility testing of yeasts approved standard. CLSI document M27-A2

[35] CLSI. 2020. Performance standards for antifungal susceptibility testing of filamentous fungi. 2nd ed. CLSI supplement M61.

[36] Hunter JD. 2007. Matplotlib: a 2D graphics environment. Comput Sci Eng 9:90–95. doi:10.1109/MCSE.2007.55

[37] CLSI. 2020. Performance standards for antifungal susceptibility testing of yeasts. 2nd ed. CLSI supplement M60.

[38] Wickham H. 2016. Data analysis, p 189–201. In ggplot2. Springer, Cham.

[39] Katoh K, Standley DM. 2013. MAFFT multiple sequence alignment software version 7: improvements in performance and usability. Mol Biol Evol 30:772–780. doi:10.1093/molbev/mst010 23329690 PMC3603318

[40] Miller MA, Pfeiffer W, Schwartz T. “The CIPRES science gateway: enabling high-impact science for phylogenetics researchers with limited resources” Proceedings of the 1st conference of the extreme science and engineering discovery environment: bridging from the eXtreme to the campus and beyond (XSEDE ’12), p 1–8. Association for Computing Machinery, New York, USA. doi:10.1145/2335755.2335836

[41] Stamatakis A. 2014. RAxML version 8: a tool for phylogenetic analysis and post-analysis of large phylogenies. Bioinformatics 30:1312–1313. doi:10.1093/bioinformatics/btu033 24451623 PMC3998144

[42] McGinnis MR, Pasarell L. 1998. In vitro testing of susceptibilities of filamentous ascomycetes to voriconazole, itraconazole, and amphotericin B, with consideration of phylogenetic implications. J Clin Microbiol 36:2353–2355. doi:10.1128/JCM.36.8.2353-2355.1998 9666022 PMC105048

[43] Espinel-Ingroff A, Cuenca-Estrella M, Cantón E. 2013. EUCAST and CLSI: working together towards a harmonized method for antifungal susceptibility testing. Curr Fungal Infect Rep 7:59–67. doi:10.1007/s12281-012-0125-7

[44] Beardsley J, Halliday CL, Chen S-A, Sorrell TC. 2018. Responding to the emergence of antifungal drug resistance: perspectives from the bench and the bedside. Future Microbiol 13:1175–1191. doi:10.2217/fmb-2018-0059 30113223 PMC6190174

[45] Arendrup MC, Prakash A, Meletiadis J, Sharma C, Chowdhary A. 2017. Comparison of EUCAST and CLSI reference microdilution MICs of eight antifungal compounds for Candida auris and associated tentative epidemiological cutoff values. Antimicrob Agents Chemother 61:e00485-17. doi:10.1128/AAC.00485-17 28416539 PMC5444165

[46] Chowdhary A, Perfect J, de Hoog GS. 2014. Black molds and melanized yeasts pathogenic to humans. Cold Spring Harb Perspect Med 5:a019570. doi:10.1101/cshperspect.a019570 25384772 PMC4526721

[47] Wu Y, Grossman N, Totten M, Memon W, Fitzgerald A, Ying C, Zhang SX. 2020. Antifungal susceptibility profiles and drug resistance mechanisms of clinical Lomentospora prolificans isolates. Antimicrob Agents Chemother 64:e00318-20. doi:10.1128/AAC.00318-20 32816726 PMC7577128

[48] Revie NM, Iyer KR, Robbins N, Cowen LE. 2018. Antifungal drug resistance: evolution, mechanisms and impact. Curr Opin Microbiol 45:70–76. doi:10.1016/j.mib.2018.02.005 29547801 PMC6135714

[49] Fisher MC, Garner TWJ, Walker SF. 2009. Global emergence of Batrachochytrium dendrobatidis and amphibian chytridiomycosis in space, time, and host. Annu Rev Microbiol 63:291–310. doi:10.1146/annurev.micro.091208.073435 19575560

[50] Romero-Zambrano GL, Bermúdez-Puga SA, Sánchez-Yumbo AF, Yánez-Galarza JK, Ortega-Andrade HM, Naranjo-Briceño L. 2021. Amphibian chytridiomycosis, a lethal pandemic disease caused by the killer fungus Batrachochytrium dendrobatidis: new approaches to host defense mechanisms and techniques for detection and monitoring. RB 6:1628–1636. doi:10.21931/RB/2021.06.01.28

[51] Prigitano A, Esposto MC, Romanò L, Auxilia F, Tortorano AM. 2019. Azole-resistant Aspergillus fumigatus in the Italian environment. J Glob Antimicrob Resist 16:220–224. doi:10.1016/j.jgar.2018.10.017 30367993

[52] Ersal T, Al-Hatmi ASM, Cilo BD, Curfs-Breuker I, Meis JF, Özkalemkaş F, Ener B, van Diepeningen AD. 2015. Fatal disseminated infection with Fusarium petroliphilum. Mycopathologia 179:119–124. doi:10.1007/s11046-014-9813-x 25234793

[53] Batista BG, Chaves M de, Reginatto P, Saraiva OJ, Fuentefria AM. 2020. Human fusariosis: an emerging infection that is difficult to treat. Rev Soc Bras Med Trop 53:e20200013. doi:10.1590/0037-8682-0013-2020 32491099 PMC7269539

[54] Nucci M, Anaissie E. 2007. Fusarium infections in immunocompromised patients. Clin Microbiol Rev 20:695–704. doi:10.1128/CMR.00014-07 17934079 PMC2176050

[55] Zhu Y, O’Brien B, Leach L, Clarke A, Bates M, Adams E, Ostrowsky B, Quinn M, Dufort E, Southwick K, Erazo R, Haley VB, Bucher C, Chaturvedi V, Limberger RJ, Blog D, Lutterloh E, Chaturvedi S. 2020. Laboratory analysis of an outbreak of Candida auris in New York from 2016 to 2018: impact and lessons learned. J Clin Microbiol 58:e01503-19. doi:10.1128/JCM.01503-19 31852764 PMC7098748

