# Supplementary material for: Evolutionary trends in antifungal resistance: a meta-analysis
Source: Microbiol Spectr. 2024 Mar 6;12(4):e02127-23. doi: 10.1128/spectrum.02127-23 (PMC10986544; doi:10.1128/spectrum.02127-23)
Supplement: Supplemental material — Figures S1 to S6. [file spectrum.02127-23-s0001.pdf]

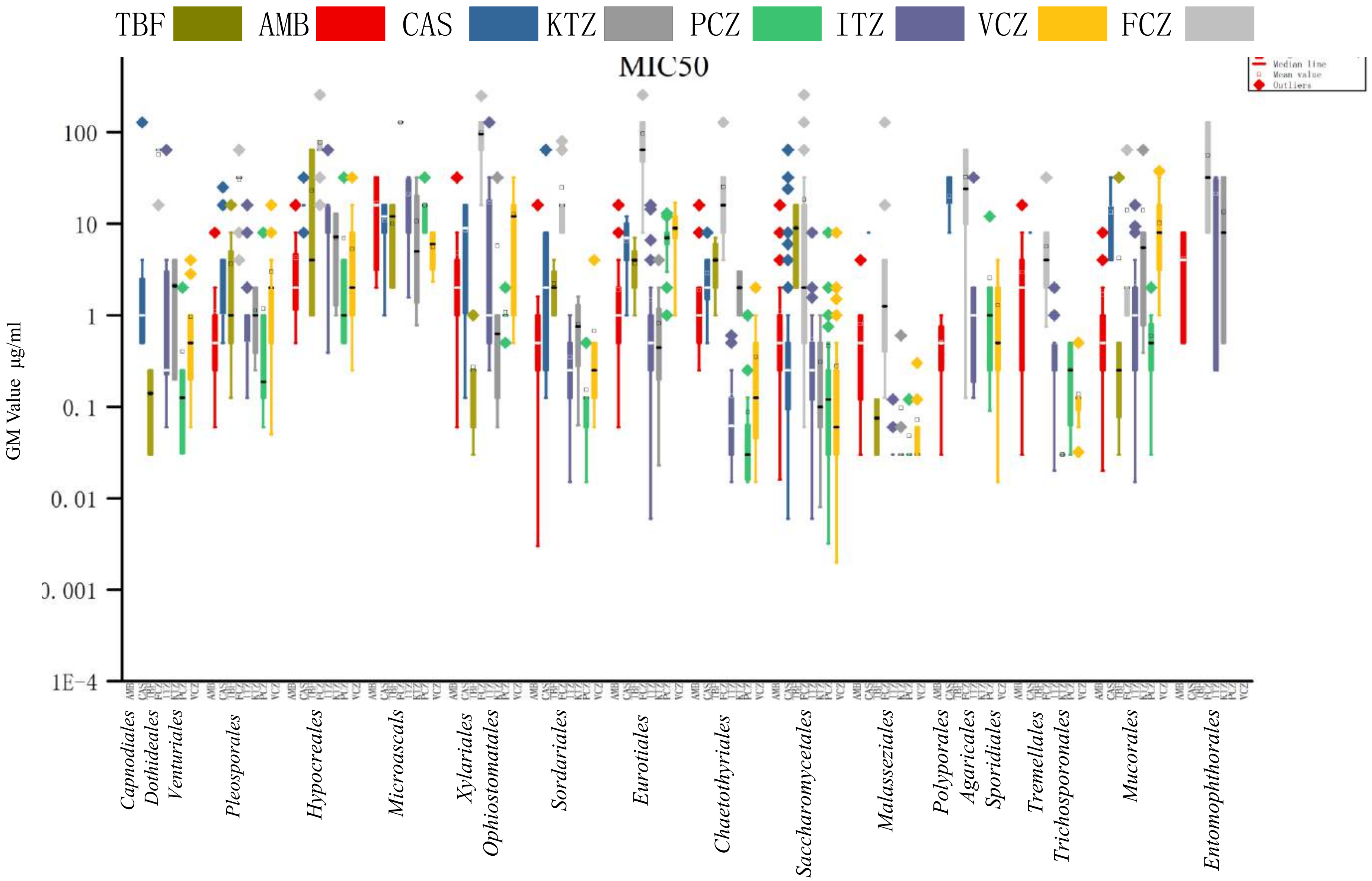

Fig. S1. Box-whisker plot MIC50

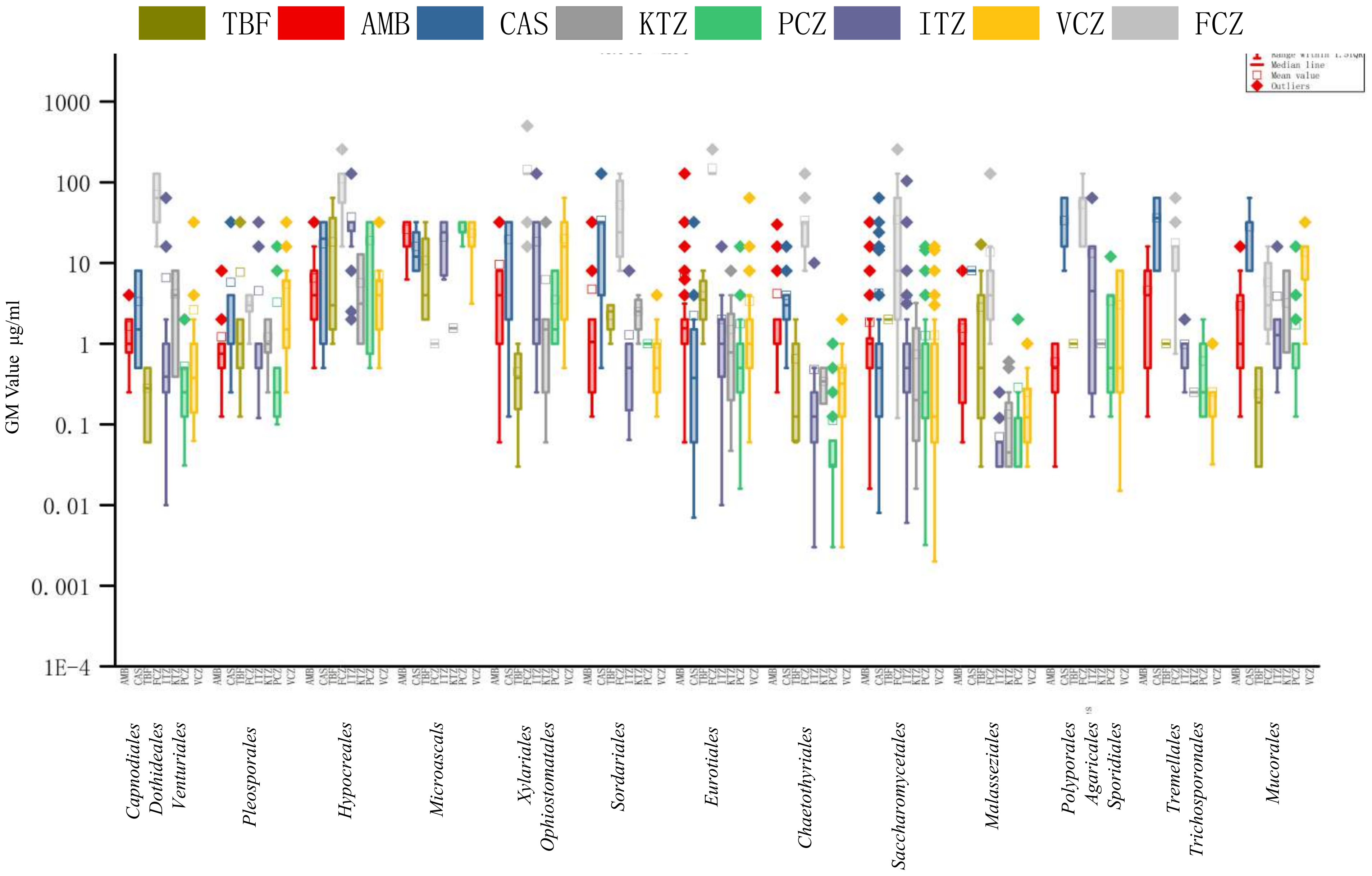

Fig. S2. Box-whisker plot MICX

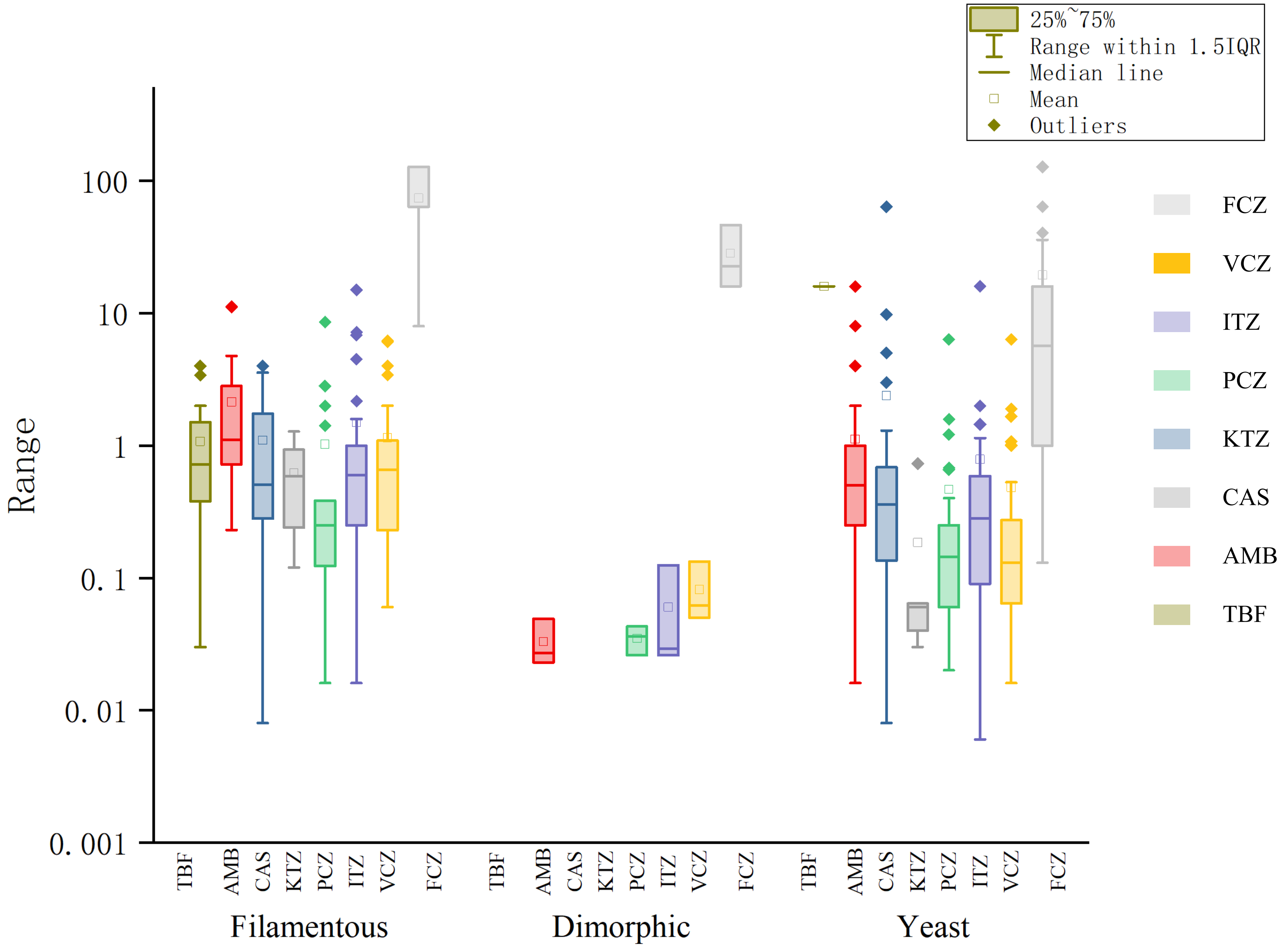

Fig. S3. Box-whisker plot-Yeast, Dimorphic, Filamentous

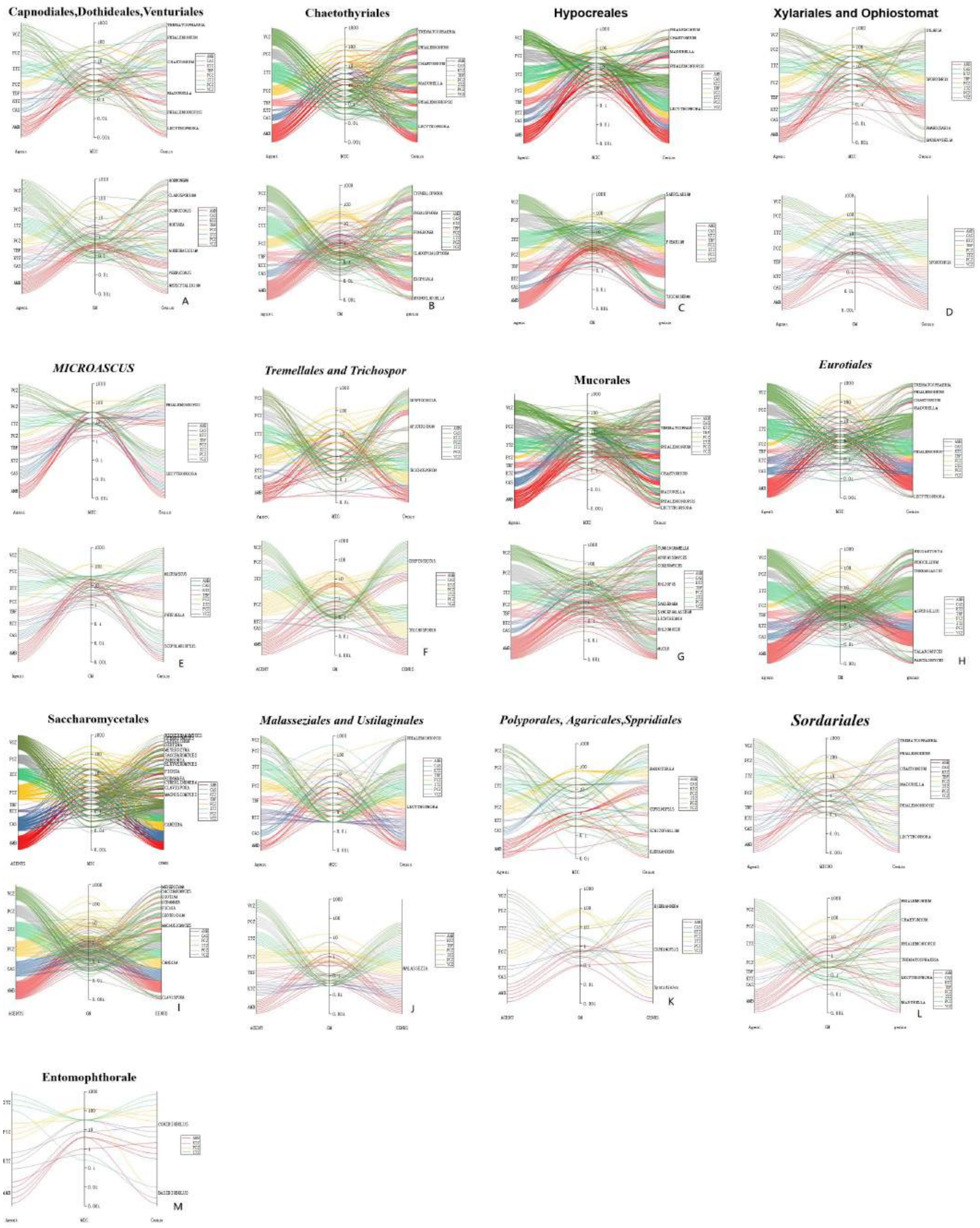

Fig. S4. Parallel coordinate plot of GM and MIC values of 15 groups.

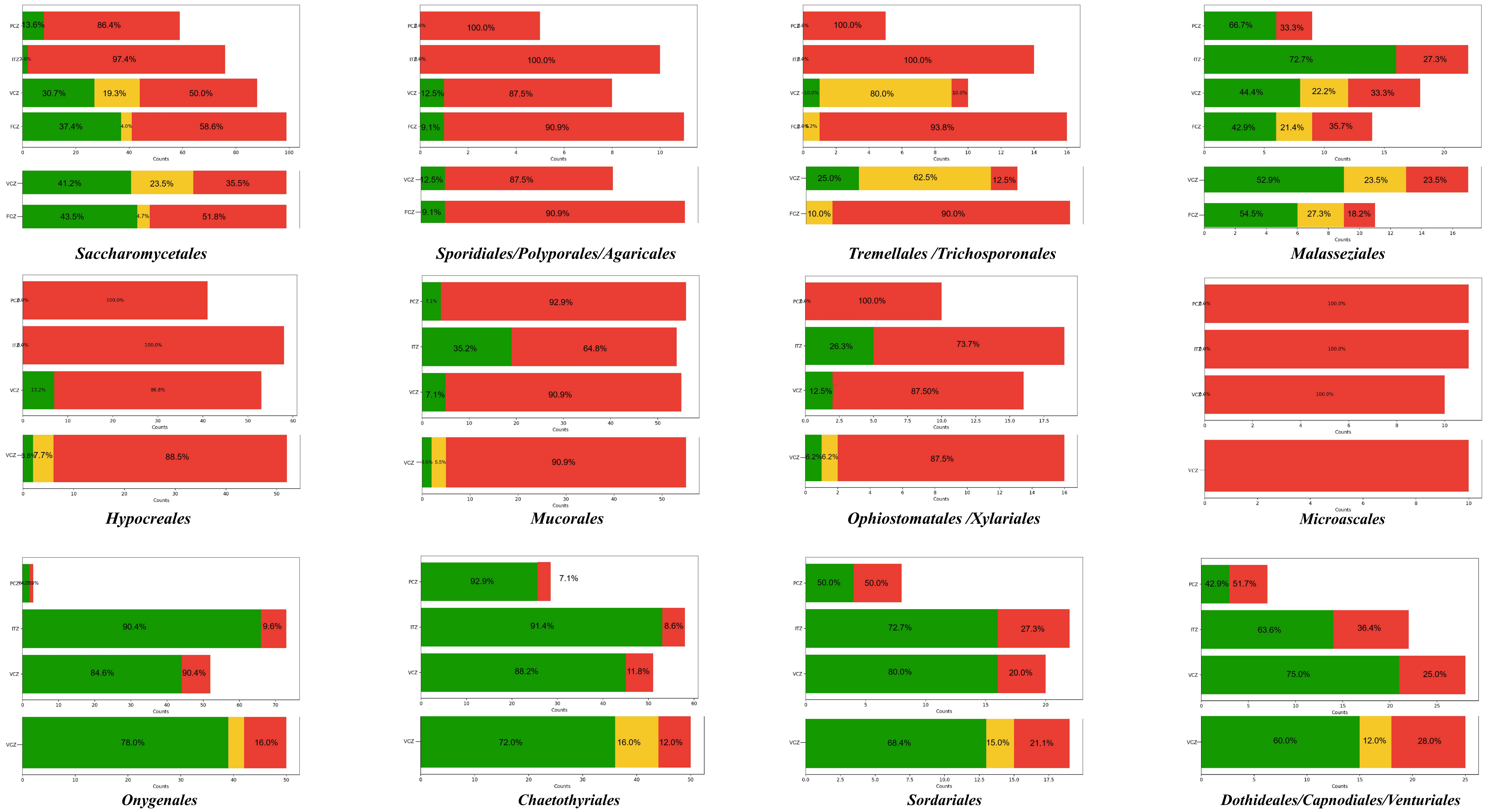

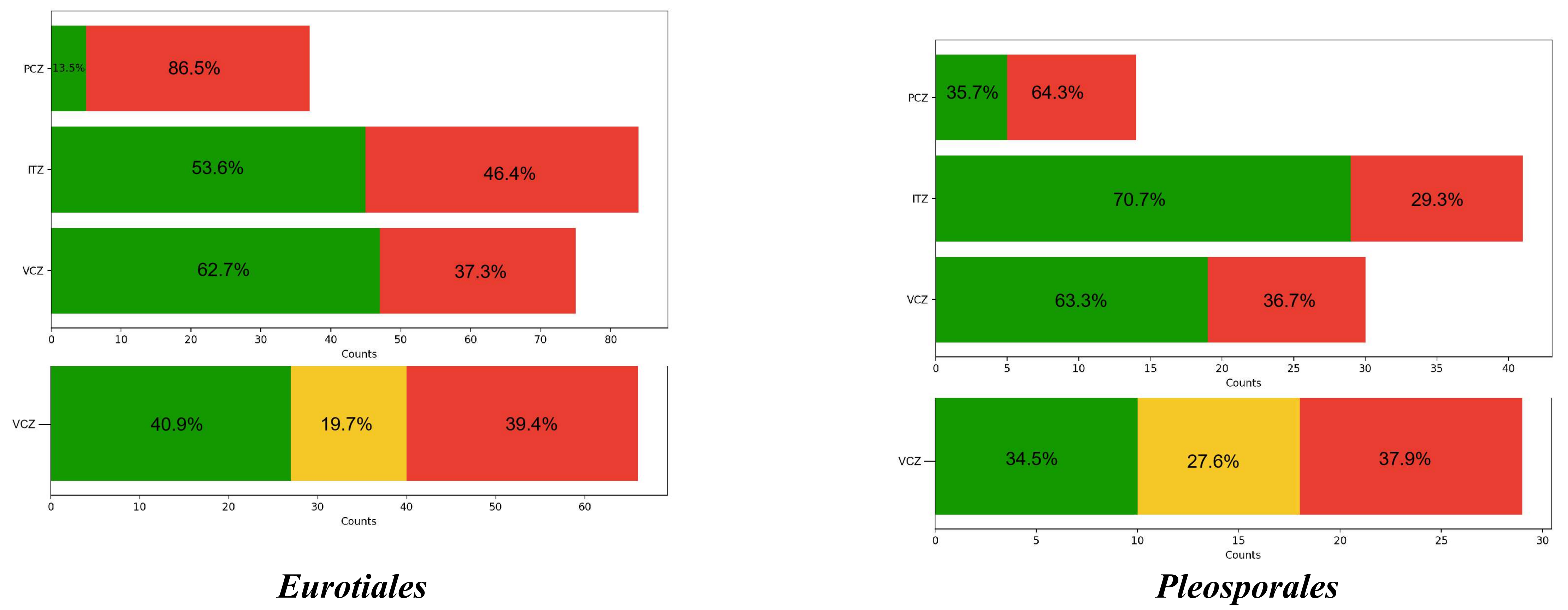

R : resistant
S : susceptible, standard dosing regimen
I : susceptible with increased exposure

Figure. S5. Two methods MIC CBP were compared using traffic light format in 15 groups (A-O; Table 1). S (susceptible, standard dosing regimen) shown in green, R (resistant) shown in red, and the buffer between these I (susceptible with increased exposure) shown in yellow. Y-axis lists the antifungal agents, from top to bottom: short-tailed azoles fluconazole (FCZ), voriconazole (VCZ), long-tailed azoles itraconazole (ITZ), posaconazole (PCZ). X-axis displays percentages of different antifungal agents in these three intervals: top = EUCAST CBP, bottom = CLSI CBP.

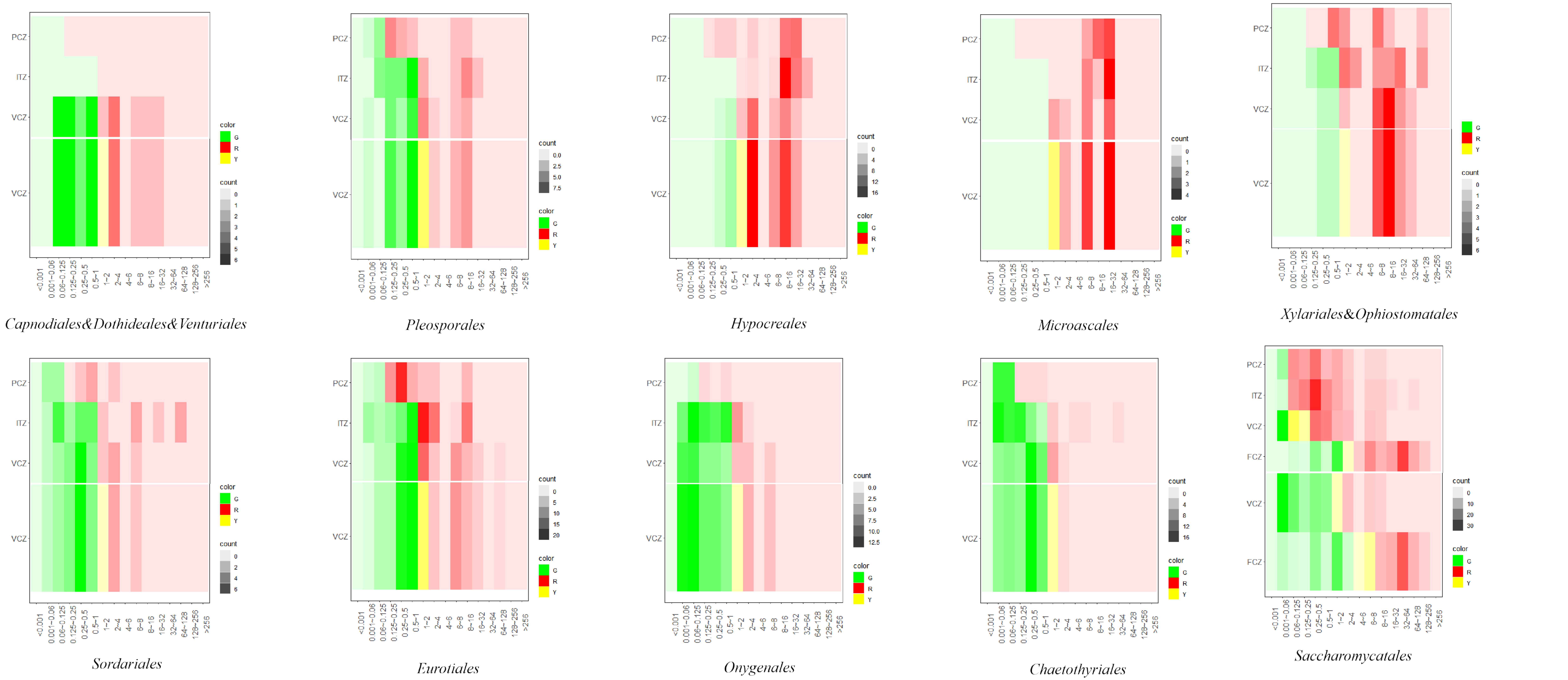

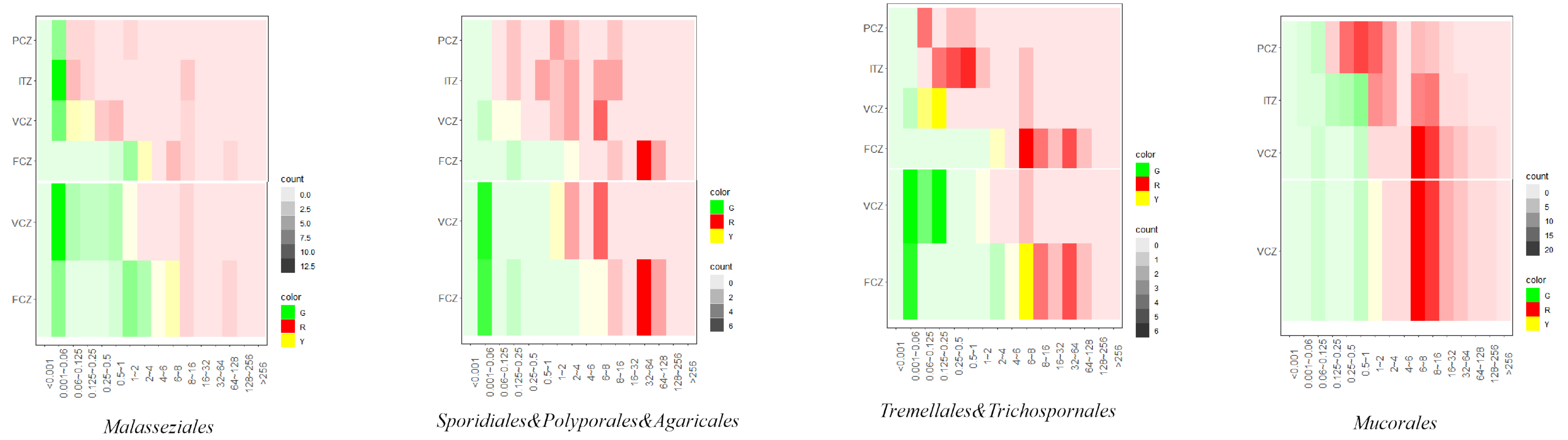

Fig S6. Fig. S6. Two methods CBP were compared in the kingdom, MICs were used. Total of 15 groups (A-O; Table 1), the top of the graphic EUCAST CBP were used, the bottom one CLSI CBP were used. Note: S (susceptible, standard dosing regimen) shown in green, R (resistant) shown in red, and the buffer between these I (susceptible with increased exposure) shown in yellow. Y-axis lists the antifungal agents from top to bottom: short-tailed azoles fluconazole (FCZ), voriconazole (VCZ), long-tailed azoles itraconazole (ITZ), posaconazole (PCZ).
